# 

**Published:** 1863-07

**Authors:** 


### BOOKS RECEIVED.

*A Practical Treatise on Fractures and Dislocations. By* Frank Hastings Hamilton, A. B., A. M., M. D., *Lt. Col. Medical Inspector, U. S. A.; Professor of Military Surgery and Hygiene and of Fractures and Dislocations in Bellevue Hospital Medical College; one of the Surgeons to Bellevue Hospital, New York; Professor of Military Surgery, etc. in the Long Island College Hospital, Brooklyn; author of a Treatise on Military Surgery. Second edition, revised and improved. Illustrated with two hundred and twenty-five wood cuts. Philadelphia:* Blalchard & Lea, 1863.

